# Cytoprotection, Genoprotection, and Dermal Exposure Assessment of Chitosan-Based Agronanofungicides

**DOI:** 10.3390/pharmaceutics12060497

**Published:** 2020-05-29

**Authors:** Farhatun Najat Maluin, Mohd Zobir Hussein, Nor Azah Yusof, Abu Seman Idris, Leona Daniela Jeffery Daim, Murni Nazira Sarian, Nor Fadilah Rajab, Siew Ee Ling, Noramiwati Rashid, Sharida Fakurazi

**Affiliations:** 1 Institute of Advanced Technology, Universiti Putra Malaysia, Serdang 43400 UPM, Selangor, Malaysia; farhatunnajat@yahoo.com (F.N.M.); mzobir@upm.edu.my (M.Z.H.); azahy@upm.edu.my (N.A.Y.); 2 Department of Chemistry, Faculty of Science, Universiti Putra Malaysia, Serdang 43400 UPM, Selangor, Malaysia; 3 Malaysian Palm Oil Board (MPOB), 6, Persiaran Institusi, Bandar Baru Bangi, Kajang 43000, Selangor, Malaysia; idris@mpob.gov.my; 4 Sime Darby Technology Centre Sdn. Bhd., UPM-MTDC Technology Centre III, Lebuh Silikon, Universiti Putra Malaysia, 1st Floor, Block B, Serdang 43400, Selangor, Malaysia; leona.daniela.jefferydaim@simedarbyplantation.com; 5 Institute of Systems Biology (INBIOSIS), Universiti Kebangsaan Malaysia, Bangi 43600 UKM, Selangor, Malaysia; murninazira@ukm.edu.my; 6 Biomedical Science Programme, Center for Healthy Aging and Wellness, Faculty of Allied Health Sciences, Universiti Kebangsaan Malaysia, Kuala Lumpur 50300, Malaysia; nfadilah@ukm.edu.my; 7 Biocompatibility Laboratory, Centre for Research and Instrumentation Management (CRIM), Universiti Kebangsaan Malaysia, Bangi 43600 UKM, Selangor, Malaysia; sieweeling@ukm.edu.my (S.E.L.); norarashid@ukm.edu.my (N.R.); 8 Department of Human Anatomy, Faculty of Medicine and Health Sciences, Universiti Putra Malaysia, Serdang 43400 UPM, Selangor, Malaysia

**Keywords:** chitosan nanoformulations, cytoprotection, genoprotection, fungicides, cellular death, dermal assessment

## Abstract

Health risks which result from exposure to pesticides have sparked awareness among researchers, triggering the idea of developing nanoencapsulation pesticides with the aim to enhance cytoprotection as well as genoprotection of the pesticides. In addition, nanocapsules of pesticides have slow release capability, high bioavailability, and site-specific delivery, which has attracted great interest from researchers. Hence, the objective of this work is to synthesize a nanoformulation of a fungicide of different sizes, namely, chitosan-hexaconazole nanoparticles (18 nm), chitosan-dazomet nanoparticles (7 nm), and chitosan-hexaconazole-dazomet nanoparticles (5 nm), which were then subjected to toxicological evaluations, including cytotoxicity, genotoxicity, cell death assay, and dermal irritation assays. Incubation of chitosan-based nanofungicides with V79-4 hamster lung cell did not reveal cytotoxicity or genotoxicity, potentially suggesting that encapsulation with chitosan reduces direct toxicity of the toxic fungicides. Meanwhile, pure fungicide revealed its high cytotoxic effect on V79-4 hamster lung cells. In addition, dermal exposure assessment on rabbits revealed that chitosan-hexaconazole nanoparticles are classified under corrosive subcategory 1C, while chitosan-dazomet nanoparticles are classified under corrosive subcategory 1B. Moreover, both chitosan-hexaconazole nanoparticles and chitosan-dazomet nanoparticles are classified as causing mild irritation.

## 1. Introduction

Pesticide covers a wide range of agrochemicals including fungicides, insecticides, herbicides, nematicides, rodenticides, or plant growth regulators. The usage pattern of pesticides can be divided into insecticides (44%), herbicides (30%), fungicides (21%), and others (5%) [1]. The main benefits of using pesticides in crop management include crop productivity, to enhance yields, to protect from excessive loss, and to increase the quantity and quality of crop production [2]. However, together with its known benefits, pesticides are inherently toxic to living organisms. Long-term or continuous exposure to pesticides via their application or substance residues (in food or drinking water) has potential negative impacts on human health [3]. Improper handling and prolonged exposure of pesticides in the agricultural industry has recorded several untoward effects of pesticide cases, including skin and eye irritation, nausea, headache, vomiting, and shortness of breath [4,5]. Meanwhile, conventional agrochemicals are well documented to impart cytotoxic and genotoxic properties following continuous exposure to farmers and land-owners.

According to Food and Drug Association (FDA, Silver Spring, MD, USA), chitosan has been approved as nontoxic and biocompatible substance [6,7]. Chitosan has cytoprotective and genoprotective effects due to its antimicrobial, antioxidant, bioadhesion, and scavenging action against free radical production [8,9]. Conventional pesticides are well documented to impart cytotoxic and genotoxic properties following continuous exposure to farmers and land-owners. Utilization of nanotechnology has allowed the pesticides to be loaded into spherical nanocapsule matrices of chitosan, which acts as a protective barrier or shell to prevent direct contact of toxic pesticides with the cell or DNA [10,11,12]. In addition, chitosan nanocapsule-based pesticides have been shown to enhance bioavailability, in addition to controlled release properties and site-specific delivery [13]. These properties are essential to ensure that the applied pesticide is delivered to the targeted site of interest instead of leaching out and run-off to the soil and nearby rivers, which may trigger health and environmental issues. These are the ideal desired properties offered by agronanopesticides for better management of crop diseases and pests. Research on the use of chitosan as a cytoprotective and genoprotective agent in pharmaceutical and agricultural fields is developing and, to date, there are limited relevant reports. Nanocapsules (hydrodynamic diameter of 400 nm) and nanoemulsions (hydrodynamic diameter of 190 nm) of chitosan loaded with triclabendazole have been reported to be able to significantly lower the cytotoxic effect of fungicides on intestinal absorptive cells compared to their counterparts [14]. In another report, chitosan-quinapyramine sulfate nanoparticles were shown to have lower cytotoxic and genotoxic effects on a HeLa cell line compared to their conventional counterparts [15]. The nanoformulations are also reported to be able to enhance the efficiency of the drug in the treatment of trypanosomes and prolong the survivability of the infected rabbit. 

The fungicides dazomet and hexaconazole, employed by the oil palm industry, serve as one of the initiatives in the management of pathogenic fungi in oil palm [7,16,17]. Nonetheless, both fungicides possess immense drawbacks as they impose a conceivable threat to the surrounding environment as well as humans. While the release of methyl isothiocyanate (MITC) from dazomet is able to extinguish *G. boninense* infection in oil palm, it is harmful towards living organisms, especially the planters, due to its high gas volatilization once in contact with water [18]. Similarly, hexaconazole has a high risk of contaminating groundwater resources due to its leaching effect [19]. 

The high efficacy of chitosan-based agronanofungicides, namely chitosan-hexaconazole nanoparticles (CHEN) [20], chitosan-dazomet nanoparticles (CDEN) [21], and chitosan-hexaconazole-dazomet nanoparticles (CHDEN) [22], as potent antifungal agents on *Ganoderma boninense*, a pathogenic fungus of basal stem rot disease in oil palm, has been proven in our previous work (in vitro [20,21,22] and in vivo [23]). The results revealed the higher antifungal activity of our newly developed agronanofungicides compared to the conventional fungicide (up to seven times) [17], as well as compared to their counterpart, pure fungicide (up to forty times) [20,21,22]. Accordingly, further evaluation on the toxicological consequences, including cytotoxicity, genotoxicity, evaluation of potential dermal irritation, and corrosion, as well as possible induction of cellular death, of our newly developed chitosan-based agronanofungicides is conducted and presented in this work. Moreover, the ability of chitosan nanocapsules as a cytoprotective agent (on 3T3 mouse fibroblast cells and V79-4 hamster lung cells) and genoprotective agent (on V79-4 hamster lung cells) are also the focus of this study. The analysis of cellular death via apoptosis and necrosis was conducted using 3T3 mouse fibroblast cells, and the dermal irritation/corrosion of chitosan-based agronanofungicides was evaluated by test patch application on the dorsal skin of rabbits. The 3T3 mouse fibroblast cells and V79-4 hamster lung cells were chosen in this study due to the possible routes of exposure of pesticide during its application, via skin contact and inhalation. We hope that the newly developed chitosan-based agronanofungicides can reduce the toxicological consequence of fungicides, and subsequently offer a sustainable alternative in the management of disease of oil palm due to *Ganoderma boninence* and to provide a safe toxicity level to the planters who may have been exposed or have come into direct contact with the fungicides. 

## 2. Materials and Methods 

### 2.1. Materials

Hexaconazole (C_14_H_17_C_l2_N_3_O, with molecular weight of 314.21 g/mol) and dazomet (C_5_H_10_N_2_S_2_, with molecular weight of 162.27 g/mol) were purchased from Aiteng (Changzhou, China) with 95% and 98% purity, respectively, and were used as received. V79-4 Chinese hamster lung cell (*Cricetulus griseus* fibroblast, CCL-93^TM^), with passage 4 and 3T3-Swiss albino ATCC ^®^ CCL-92™ Mus musculus embryo were bought from American Type Culture Collection (Manassas, VA, USA). Low melting points agarose (LMA) and normal melting point agarose (NMA), acetone, uranyl acetate, hydrogen peroxide, ethidium bromide solution and 3-(4,5-Dimethyl-2-thiazolyl)-2,5-diphenyl-2H-tetrazolium bromide (MTT) reagent were obtained from Sigma Aldrich (St. Louis, MO, USA). Dubelcco Modified Eagle Media (DMEM), fetal bovine serum, penicillin–streptomycin, sodium cacodylate buffer solution, glutaraldehyde, and phosphate buffer saline were purchased from Gibco–Fisher Scientific (England, UK). Dimethyl sulfoxide, osmium tetroxide, and phosphate-buffered (PBS) saline were procured from Merck (Darmstadt, Germany). Propidium iodide staining solution, annexin V binding buffer and FITC-Annexin V apoptosis staining/detection kit were bought from BD Bioscience (San Jose, CA, USA). Copper grids (3 mm, 200 mesh) were acquired from Agar Scientific (Essex, UK).

### 2.2. Preparation of Chitosan-Based Agronanofungicides

CHEN, CDEN, and CHDEN were formulated using the method described earlier [20,21,22] and the details of the materials are listed in Table 1. The fungicides (hexaconazole and/or dazomet) were loaded into the nanocapsule of chitosan using an ionic gelation method. The crosslinking agent, sodium tripolyphosphate (TPP), plays an important role in producing the small size of the nanoparticles. The nanoparticles were obtained through the electrostatic interaction between the negatively charged TPP and the positively charged chitosan embedded with the fungicide. The TPP-to-chitosan ratio in all nanoformulations was fixed at 1:2.5 (*v*/*v*), which is believed to be the suitable ratio for the preparation of nanoparticles in this work [20]. The stabilizing agent, TWEEN-80, has also been added to prevent aggregation of the nanoparticles. Prior to the high-resolution transmission electron microscopy (HRTEM) measurement, the nanoparticles were suspended in deionized water and sonicated for 5 min at 70 watts. The test substance was prepared by dissolving 10 mg/mL of active ingredient in 10% acetone via sonication for 15 min. The solution was then vortexed and serially diluted into 1.00, 0.50, 0.25, 0.1, 0.06, 0.03, and 0.01 mg/mL and kept in a freezer at −20 °C prior to analysis.

### 2.3. In Vitro Cytotoxicity and Genotoxicity Analysis

#### 2.3.1. Cell Culture Conditions

V79-4 hamster lung cells were cultured in Dulbecco’s Modified Eagle’s Medium (DMEM) supplemented with 10% (*v/v*) fetal bovine serum, 1 mM sodium pyruvate, and 1% antibiotics (containing 100 unit/mL penicillin and 100 μg/mL streptomycin). Fibroblast cells, 3T3, were seeded into six-well plates and cultured at 37 °C in a humidified atmosphere of 5% carbon dioxide and 95% air. 

#### 2.3.2. Cell Viability 3-(4,5-Dimethyl-2-thiazolyl)-2,5-diphenyl-2H-tetrazolium Bromide (MTT) Assay

The cell viability was detected by MTT (3-(4,5-Dimethylthiazol-2-yl)-2,5-diphenyltetrazolium bromide) assay. The colorimetric test of MTT salt was used to quantify the ability of dehydrogenase enzymes of mitochondria to cleave in an active cell and subsequently convert the yellow salt to formazan blue. Briefly, 100 µL of V79-4 hamster lung cells were seeded onto 96-well culture plates at 1.0 × 10^5^ cells per well and incubated overnight to allow cell attachment. Then, cultured cells were replaced with medium containing pure hexaconazole, pure dazomet, CHEN, CDEN, and CHDEN in various concentrations of active ingredient ranging from 0.01 μg/mL to 1.00 mg/mL. The control cells were not exposed to fungicide and chitosan-fungicide nanoparticles. At specific time points of 24, 48, and 72 h of incubation, 20 μL MTT reagents were added to each well and incubated for 4 h. Then, 100 µL of dimethyl sulfoxide was added to dissolve blue formazan. The number of viable cells was measured at 570 nm using a Perkin Elmer microplate reader (Shelton, CT, USA). The percentage of cell viability was calculated using Equation (1) [24]:

(1) $$Percentage\;of\;cell\;viability\;\left(\%\right)=\frac{\left(Abs\;sample-Abs\;blank\right)}{Abs\;control-Abs\;blank}\times 100$$

#### 2.3.3. Alkaline Comet Assay

The DNA damage was estimated using an alkaline comet assay and carried out using a previously published method [25]. Growth medium, 1 mM hydrogen peroxide, and 1% v/v acetone served as a negative control (NC), positive control (PC), and vehicle control (VC), respectively. Seeded V79-4 hamster lung cells were exposed in the growth medium containing 2 mL of CHEN and CDEN (1 mg/mL of active ingredient) for 24 h, while cell exposure was done at 30 min for PC and 24 h for NC and VC. Two slides of low melting point agarose (LMA) and normal melting point agarose (NMA) were then prepared from each well. Then, the slides were transferred into a Coplin jar containing a chilled lysis buffer. To remove all cellular contents, the lysis process was performed at 4 °C for 1 h. After lysis, the slides were subjected to DNA unwinding for 20 min by immersing with electrophoresis buffer at 20–25 V and 300–350 mA for 20 min. Once finished, the slides were washed with neutralization buffer and stained with ethidium bromide solution. The resulting slides were then analyzed under a fluorescent microscope using Comet Assay III analysis software (Trevigen, MD, USA).

### 2.4. Cellular Death Assay

#### 2.4.1. Cell Culture Conditions

3T3 mouse fibroblast cells were cultured in Dulbecco’s Modified Eagle’s Medium (DMEM) supplemented with 10% fetal bovine serum (FBS) and 1% antibiotics (containing 100 unit/mL penicillin and 100 μg/mL streptomycin). Fibroblast cells were cultured at 37 °C in a humidified atmosphere of 5% carbon dioxide and 95% air.

#### 2.4.2. FITC-Annexin V/PI Apoptosis Detection

For the mode of cell death detection assay, FITC-Annexin V stain was used to investigate phosphatidylserine externalization in apoptosis and propidium iodide stain to detect necrotic cells. Approximately 2.5 × 10^5^ 3T3 mouse fibroblast cells were seeded in a T-25 flask with 5 mL of CHEN and CDEN at 1 mg/mL of active ingredient. After 48 h of incubation, the cultures were washed twice with cold phosphate-buffered saline followed by the cell detachment. The cells were then centrifuged at 1000 rpm for 5 min and resuspended using Annexin V binding buffer. Then, the cells were stained using FITC-Annexin V and propidium iodide for 15 min [24]. The evaluation of apoptosis and necrosis was assessed using a flow cytometer, LSR-Fortessa, BD Bioscience (San Jose, CA, USA). Data acquisition and analysis were performed using Facs Diva version 6, BD Bioscience (San Jose, CA, USA).

#### 2.4.3. Cell Morphology

The visualization of cell morphology exposed to the test substance was performed using high-resolution transmission electron microscopy (HRTEM). Briefly, 3T3 mouse fibroblast cells were grown in a T-75 flask until 80% confluence; then, the cells was exposed to CHEN and CDEN at 1 mg/mL of active ingredient for 48 h. At the end of the incubation, the cells were washed twice with cold phosphate-buffered saline, detached from the T-75 flask by scraping, and centrifuged at 1000 rpm for 5 min. Then, the cell pellets were fixed in 4% cacodylate-glutaraldehyde buffered for up to 6 h, washed in cacodylate buffer thrice for 10 min. Samples were postfixed in 1.3% osmium tetroxide in 0.2 M cacodylate buffer for 2 h and dehydrated in graded acetone (35%, 50%, 75%, 95%, and 100%) and embedded in 50% (*w/w*) epoxy embedding resin. Blocks were cured for 48 h at 60 °C before thin sections (70–80 nm) were cut using an ultramicrotome Leica Microsystems (Wetzlar, Germany) and mounted on copper grids (3 mm, 200 mesh). Grids were stained for 75 min in saturated uranyl acetate solution, then for 100 s in lead citrate, examined, and photographed with a Philips CM10 high-resolution transmission electron (HRTEM) microscope (Eindhoven, Netherlands) combined with a Soft Imaging system to process and analyze the micrographs.

### 2.5. Dermal Corrosion/Irritation Assay

#### 2.5.1. Animals, Materials, and Experimental Design

New Zealand white rabbits with body weight in the range of 3–5 kg were purchased from Sapphire Enterprise, Selangor, Malaysia, and Pizhou Dong Fang, Ltd. China. A total of six rabbits (two males and four females) were housed individually in standard stainless-steel cages with mesh floors and acclimated as recommended in animal husbandry guidelines. For each test material, one male rabbit was used for the initial test and followed by two female rabbits as the confirmatory test. Only healthy animals with no pre-existing skin irritation were selected for this study. The rabbits were reared on a diet using standard rabbit pellets and kept in a room at 18–23 °C, with a relative humidity of 51.2–56.2% and 12 h light/dark cycles (7:00–19:00 and 19:00–7:00, respectively). 

The experiments were conducted at the Biocompatible Laboratory, Universiti Kebangsaan Malaysia (Bangi, Malaysia). The study design was performed in compliance with the appropriate provision of the Organization for Economic Cooperation and Development (OECD) guideline for testing of chemicals TG 404 and the Globally Harmonized System of Classification and Labelling Chemicals (GHS) [26,27]. The management of the study, including quality assurance procedures, was conducted in compliance with Malaysian Standard (MS ISO/IEC 17025) Accreditation and Specific Technical Requirement 1.2 (STR 1.2). All procedures involving the use and care of animals adhere to approval from Universiti Kebangsaan Malaysia’s Animal Ethics Committee (BIOSERASI/UKM/2019/MIMINORHILDA/5-JULY/1014-JULY-2019-DEC-2019, 5 July 2019). 

#### 2.5.2. Experimental Procedure

CHEN and CDEN were moistened with a minimum amount of phosphate buffer saline to prepare a paste, producing CHEN-patch and CDEN-patch for the dermal application. An area of 6 cm^2^ in the dorsal body of each side of the rabbit was clipped free of hair with a clipper. Care was taken to avoid abrading the skin and trauma. Upon application, 0.5 g CHEN and CDEN patches were directly applied on the designated site using 3 × 2 cm absorbent gauze and wrapped with a surgical tape and elastic bandage to avoid dislocation of the gauze pad and to minimize evaporation. The adjacent area of the untreated skin served as a negative control.

For the initial test, a total of three patches were applied sequentially to the same designated dorsal area of the rabbit skin, in which the first, second, and third patches were removed after 3 min, 1 h, and 4 h, respectively. For the confirmatory test, a patch of test materials was applied for 4 h to the two rabbits in a sequential manner. Next, the residual test materials were removed with filtered water and the response was graded and scored as in Table 2 immediately upon each patch’s removal and at 1, 24, 48, and 72 h after patch removal. Other adverse changes on the skin sites were recorded. 

### 2.6. Statistical Analysis

All data were expressed as means ± standard deviation (SD). The statistical significance was assessed by one-way ANOVA followed by Tukey’s test for multiple comparisons for all tests. A *p*-value less than or equal to 0.05 (*p* ≤ 0.05) was considered statistically significant.

## 3. Results

### 3.1. In Vitro Cytotoxicity Analysis

**Figure 1:**
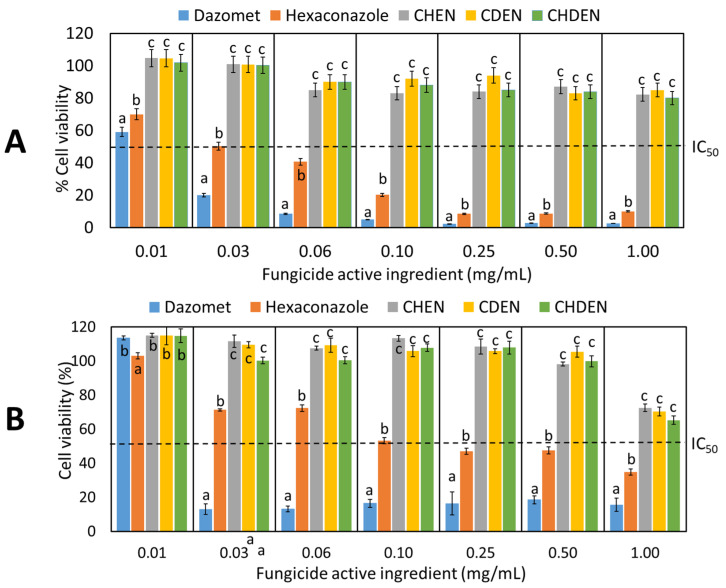
In vitro cell viability of (**A**) V79-4 hamster lung cells and (**B**) 3T3 mouse fibroblast cells treated with pure fungicide and chitosan-based agronanofungicides, where different letters (a, b, c) in the same concentrations indicate significant differences between means (*p* ≤ 0.05) according to Tukey’s test; the error bars represent the standard deviation of the mean.

As shown in Figure 1, the viability of V79-4 hamster lung and 3T3 mouse fibroblast cells exposed to pure hexaconazole (0.03 and 0.10 mg/mL, respectively) and pure dazomet (0.03 and 0.03 mg/mL, respectively) were less than the viability seen with IC_50_ (50% inhibitory concentration), which indicates their high cytotoxicity. On the other hand, CHEN, CDEN, and CHDEN show a significant improvement with satisfactory biocompatibility where the proliferation of V79-4 hamster lung cell remained above IC_50_ (half maximal inhibitory concentration) at 83%, 85%, and 80% viability, respectively, up to 1 mg/mL. The 3T3 mouse fibroblast cells exposed to CHEN, CDEN, and CHDEN also showed a significant improvement which remained above IC_50_ (half maximal inhibitory concentration) at 73%, 70%, and 65% viability, respectively, up to 1 mg/mL. There was no significant difference in the cell viability following treatment with all three formulated chitosan-based agronanofungicides in all the tested concentrations. In conclusion, cytotoxicity analysis established that chitosan was capable of reducing the toxic level of the fungicides hexaconazole and dazomet in the final product of CHEN, CDEN, and CHDEN. 

### 3.2. In Vitro Genotoxicity Analysis

The genotoxic effect of CHEN and CDEN was quantified using the tail moment value, which indicates the breaking and damage of single and double DNA strands. The value was calculated using Equation (2) and analyzed using Comet Assay III analysis software, where a ≤ 5 value indicates no DNA damage, while a value of > 5 indicates DNA damage [28]. As listed in Table 3, V79-4 hamster lung cells exposed to both CHEN and CDEN produced tail moment values below 5, indicating no broken or damaged DNA. No significant difference in the tail moment value of both CHEN and CDEN was observed with the negative control. Hence, it can be interpreted that both chitosan-based agronanofungicides were nongenotoxic in the tested cell line at 1 mg/mL. To validate the method, growth medium, 1 mM hydrogen peroxide, and 1% (*v/v*) acetone, which served as a negative control (NC), positive control (PC), and vehicle control (VC), respectively, were performed simultaneously, and the PC showed high DNA damage with a tail moment value of 12.93. The vehicle control refers to the solvent used in the preparation of the nanoparticles prior to the alkaline comet assay.

(2) Tail moment value=% DNA in tail×tail length  

### 3.3. Cellular Death Assay

In these studies, the cellular death mechanism of 3T3 mouse fibroblast cells was analyzed to identify the possible mode of cell death induced by the material as either apoptosis or necrosis. The apoptosis mechanism involves endonuclease activation, which causes internucleosomal DNA breaks, whereas necrosis involves membrane damage [27]. Apoptosis proceeds through two main stages, i.e., early and late apoptosis, characterized by the changes in cell morphology. After introduction of the “enemy”, early apoptosis begins with DNA damage, followed by the loss of mitochondrial membrane integrity. At the late phase of apoptosis, shrinkage of the cell membrane is observed. In this work, we define the apoptosis mechanism as cell death progression occurring in each treatment over time (48 h). As shown in Table 4, after 48 h of incubation, pure dazomet showed the highest percentage of cell death, with 85.5% of cells in late apoptosis and 10.2% in early apoptosis. Only 4.2% of viable cells survived. After the 48 h treatment of dazomet encapsulated by chitosan (CDEN), the viable healthy cells were significantly increased to 34.5%, resulting in the reduction of late apoptosis to only 12.2%. Therefore, the induction of cell death by CDEN at 48 h of incubation was through early apoptosis (52.4%), meaning it causes slower cell death progression. Moreover, pure hexaconazole showed signs of cell death with 49.1%, 3.2%, and 0.7% of cells in early apoptosis, late apoptosis, and necrosis, respectively. Meanwhile, the healthy viable cells were recorded at 47.0%. A significant increase in viable healthy cells (56.4%) can be observed with the 3T3 mouse fibroblast cells treated with CHEN, leading to a significant decrease in cells undergoing early apoptosis and late apoptosis, at 28.7% and 4.7%, respectively. In addition, no significant difference in necrosis cells (≤ 1%) was observed in all treatments.

**Figure 2:**
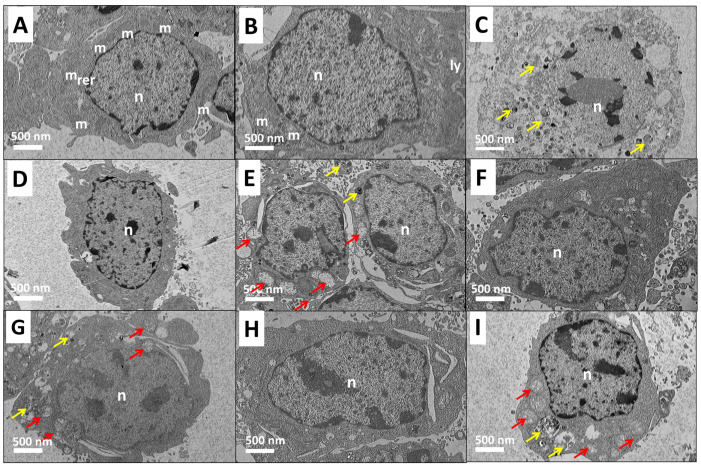
High-resolution transmission electron microscopy (HRTEM) micrographs of 3T3 mouse fibroblast cells of control (**A**) at 0 h, (**B**) at 48 h, and treated groups of 1 mg/mL active ingredient of (**C**) late apoptosis of dazomet (**D**) healthy cell of hexaconazole, (**E**) early apoptosis of hexaconazole, (**F**) healthy cells of CHEN, (**G**) early apoptosis of CHEN, (**H**) healthy cells of CDEN, and (**I**) early apoptosis of CDEN, at 48 h exposure, where n is a nucleus, m is mitochondria, ly is lysosome, and rer is rough endoplasmic reticulum. The red arrow indicates swollen mitochondria and the yellow arrow indicates condensed vacuoles. Magnification 1500×.

The analysis of cellular death was then further visualized using HRTEM (Figure 2). The micrograph of the control (nontreated 3T3 fibroblast cells) shows an intact cellular and nuclear membrane with a smooth outlined nucleus and lysosome. The mitochondria and rough endoplasmic reticulum maintained their integrity (Figure 2A,B). In addition, early apoptosis characteristics, such as increased condensed vacuole formation (yellow arrow) and pyknosis (condensation of chromatin in the nucleus of a cell) [29,30], were observed in all the treated groups (Figure 2C–F). Pure dazomet also showed a disruption of membrane structure, which may lead to late apoptosis and necrosis [31]. Meanwhile, the cells treated with hexaconazole, CHEN, and CDEN have shown no disruption of cell membrane structure. Hence, both analyses confirmed the faster cell death progression of pure dazomet in 3T3 mouse fibroblast cells compared to pure hexaconazole, CHEN, and CDEN. 

### 3.4. Dermal Irritation/Corrosion Analysis 

As described earlier, the study was initiated using one rabbit with application of one single dose (0.5 g paste) per test material (CHEN and CDEN). The initial test on dermal irritation was performed by applying the test patch three times. The exposure of each test patch on the rabbit skin was performed sequentially only if no severe skin reaction was observed. However, the exposure was only be extended up to 4 h if the finding at each application suggested that it was humanely appropriate to extend the exposure.

Table 5 shows the skin reaction scores upon the 4 h exposure of CHEN and CDEN on the dorsal skin of the rabbit based on the grading of the skin reactions (Table 2). The study was stopped at 24 h for CHEN because severe skin reaction was observed. After 24 h, the CHEN patch was removed and the dorsal skin of the rabbit showed signs of a corrosive lesion with a 5 mm brownish line typical of skin erosion. In addition, at 48 h after removal of the CDEN-patch, the rabbit skin showed a sign of corrosion lesion with slight erosion. Hence, the study was terminated at 48 h.

Moreover, erythema and edema formations were observed in both applications of CHEN and CDEN patches from the immediate patch removal until the end of the study. Moreover, to confirm the irritation response, two additional rabbits were used per test material of CHEN and CDEN. The test patch was applied once with 4 h of exposure, and the results on the skin reaction scores of the confirmatory test are shown in Table 5. For both CHEN and CDEN, skin irritation was observed immediately and at 1 h after removal of the test patch, respectively. Hence, the response was confirmed, and the study was terminated before the skin conditions became worse. 

Furthermore, to determine the corrosion and irritation category of CHEN and CDEN, the skin reaction scores were then further categorized according to the Globally Harmonized System of Classification and Labelling Chemicals (GHS), as in Table 6 [27]. Hence, CHEN is classified under corrosive subcategory 1C, while CDEN is classified under corrosive subcategory 1B. Both CHEN and CDEN are classified as causing mild irritation. 

## 4. Discussion

Pure dazomet is an acutely toxic substance with the highest toxicity compared to other tested materials. The 24 h exposure of pure dazomet on the V79-4 hamster lung cells yielded IC_10_ and IC_25_ values of 0.06 and 0.03 mg/mL, respectively. Meanwhile, only 12.9% of 3T3 mouse fibroblast cells survived upon exposure to 1.0 mg/mL of pure dazomet. In addition, 85.5% of the 3T3 mouse fibroblast cells underwent late apoptosis after exposure to pure dazomet for 48 h. The HRTEM images also revealed the rupture of the cell membrane, indicating late apoptotic or necrotic cell death [31]. In addition, according to the World Health Organization (WHO, 2010), dazomet is classified as a Class II moderately hazardous material with LD_50_ (lethal dose, 50%) of 640 mg/kg [32]. Moreover, according to the European Food Safety Authority (EFSA, 2010), the acceptable daily intake (ADI) and acceptable operator exposure level (AOEL) of dazomet was established at 0.01 mg/kg of body weight/day and 0.015 mg/kg of body weight/day, respectively [33]. Moreover, it is known that the high toxicity of dazomet is due to its gaseous degradation of methyl isothiocyanate (MITC). Hence, to avoid any health risk, Dourson et al. suggested that the minimum airborne exposure of MITC is 0.2 mg/mL for 4 h of exposure and 0.8 mg/mL for 14 min of exposure [34]. It is also reported that dazomet may cause skin sensitization, eye irritation, and respiratory problems [35,36].

Pure hexaconazole was found to be highly toxic to V79-4 hamster lung cells, with IC_10_ and IC_25_ values of 0.25 and 0.10 mg/mL, respectively. In 3T3 mouse fibroblast cells, only 34.8% of cells were viable after treatment with 1 mg/mL hexaconazole for 24 h. In addition, half of the 3T3 mouse fibroblast cells underwent early apoptosis after 48 h of exposure to pure hexaconazole. In addition, according to WHO (2010), hexaconazole is classified under Class III; a slightly hazardous substance, with an LD_50_ of 2180 mg/kg [32]. Moreover, the ADI and the no-observed-effect level (NOEL) of hexaconazole was established at 0.005 mg/kg of body weight/day and 0.5 mg/kg of body weight/day, respectively [37]. It is also reported that prolonged exposure to hexaconazole may cause skin irritation and respiratory problems [38]. 

Surprisingly, the formulated chitosan-based agronanofungicides, CHEN, CDEN, and CHDEN, showed a low cytotoxic effect on 3T3 mouse fibroblast cells and V79-4 hamster lung cells, with viability remaining above 65% at the highest tested concentration (1 mg/mL), after 24 h exposure. Moreover, CHEN and CDEN also showed a nongenotoxic effect on the V79-4 hamster lung cells after 24 h exposure. However, early apoptosis (28.7%) was found in the 3T3 mouse fibroblast cells treated with CHEN for 48 h, whereas early apoptosis (52.4%) and late apoptosis (12.2%) were found in 3T3 mouse fibroblast cells treated with CDEN for 48 h. The result is in agreement with the work done by Chauhan et al., where the chitosan-hexaconazole nanocapsule reduced the cytotoxic effect on the Vero cell line compared to their commercial formulation and free hexaconazole [39]. In another work, Marayuma et al. reported that the chitosan nanoencapsulation of herbicides (imazapic and imazapyr) reduced the cytotoxic and genotoxic effect on a hamster ovary cell line compared to free herbicides [40], hence proving the ability of chitosan nanocapsules as cytoprotective and genoprotective agents against toxic pesticides. As described earlier, the nanocapsule of nontoxic and biocompatible chitosan was able to act as a protective wall and subsequently shielded the toxic effects of pesticides. The controlled release properties of chitosan-based agronanofungicides (proved in our previous work [20,21,22]) might also contribute to the cytoprotection and genoprotection effect since the fungicides are gradually released in a sustained manner, instead of being released at the same time. Moreover, studies have shown that chitosan (without any modification) exhibits no side effects on human oral administration at 4.5 g/day. Their LD_50_ was established at 16 g/kg body weight, which is equivalent to the household intake of sugar or salt [6]. Due to some circumstances, no data on genotoxicity, cellular death mechanism, and dermal exposure assessment of CHDEN can be provided. However, we believe that CHDEN might also be nongenotoxic in V79-4 hamster lung cells. This is because CHDEN exhibits a similar cytotoxic response to CHEN and CDEN. CHDEN after 48 h of incubation might induce both of early and late apoptosis due to the combination of both fungicides in the nanoparticles. As previously published, CHDEN exhibits slower and longer release time with a half-life (t_1/2_) of 53 and 15 h for hexaconazole and dazomet, respectively, while the t_1/2_ of hexaconazole and dazomet in CHEN and CDEN, respectively, were 42 and 11 h, respectively [20,21,22]. This controlled release of CHDEN might also contribute to reducing the toxic effect of fungicides.

In addition, both CHEN and CDEN showed mild corrosion and irritation on the rabbit skin after 24 and 48 h exposure, respectively. Moreover, we believe that CHDEN might also cause mild corrosion and irritation on the rabbit skin. The results indicated that the synthesized chitosan-based agronanofungicides are safe to be used as alternative solutions in crop disease management, as long as the safety precautions, such as wearing gloves, are taken during chemical handling.

## 5. Conclusions

In summary, the toxicological consequences of the three newly developed chitosan-based agronanofungicides were evaluated. Cytotoxicity (on 3T3 mouse fibroblast cells and V79-4 hamster lung cells) and genotoxicity studies (on V79-4 hamster lung cells) revealed a cytoprotective and genoprotective effect of the chitosan-based agronanofungicides (up to 1 mg/mL) over the fungicides at 24 h exposure. Pure dazomet and pure hexaconazole had strong cytotoxic effects on V79-4 hamster lung cells, with only 10% and 3% cell viability observed at 1 mg/mL. The cellular death assay revealed the higher number of healthy cells found in the cells treated with chitosan-based agronanofungicides compared to pure fungicide. In addition, formulated chitosan-based agronanofungicides exhibited mild corrosion and irritation of rabbit skin after prolonged exposure at 24 h (chitosan-hexaconazole nanoparticles) and 48 h (chitosan-dazomet nanoparticles), indicating that necessary precaution must be taken while handling these chemicals.

## Figures and Tables

**Table 1:** Pure fungicide and formulated chitosan-based agronanofungicides.

Descriptions	Composition * (% *w/w*)	Nanoparticle Diameter ** (nm)	Appearance	Abbreviations
Pure hexaconazole	H (95%)	-	Yellowish-white powder	Hexaconazole
Pure dazomet	D (98%)	-	White powder	Dazomet
Chitosan-hexaconazole nanoparticles	CS (85%) H (15%)	18	Yellowish-white powder	Chitosan-hexaconazole Nanoparticles (CHEN)
Chitosan-dazomet nanoparticles	CS (57%) D (33%)	7	Yellowish-white powder	Chitosan-dazomet Nanoparticles (CDEN)
Chitosan-hexaconazole-dazomet nanoparticles	CS (86%) H (7%) D (7%)	5	Yellowish-white powder	Chitosan-hexaconazole-dazomet Nanoparticles (CHDEN)

* H is for hexaconazole, D is for dazomet, and CS is for chitosan ** measured using high-resolution transmission electron microscopy (HRTEM).

**Table 2:** Grading of the skin reactions.

Reactions	Descriptions	Score
Erythema and eschar formation (E)	No erythema	0
	Very slight erythema (barely perceptible)	1
	Well-defined erythema	2
	Moderate to severe erythema	3
	Severe erythema (beef-redness) to eschar formation	4
Edema formation (O)	No edema	0
	Very slight edema (barely perceptible)	1
	Slight edema	2
	Moderate edema (raised more than 1 mm)	3
	Severe edema (raised more than 1 mm and extending beyond exposure area)	4

**Table 3:** DNA damage parameters derived from the alkaline comet assay, where different superscript letters in the same row (a, b, c) indicate significant differences between means (*p* ≤ 0.05) according to Tukey’s test; the values after ± represent the standard deviation of the mean.

DNA Damage Parameters	Negative Control (NC)	Positive Control (PC)	Vehicle Control (VC)	CHEN	CDEN
Average tail DNA (%)	0.02 ± 0.00 ^a^	0.15 ± 0.03 ^b^	0.03 ± 0.01 ^a^	0.02 ± 0.01 ^a^	0.01 ± 0.01 ^a^
Average tail length	23.75 ± 3.28 ^a^	86.61 ± 3.97 ^b^	17.48 ± 1.22 ^c^	19.60 ± 1.65 ^c^	19.87 ± 1.88 ^c^
Average tail moment value	0.53 ± 0.09 ^a^	12.93 ± 0.51 ^b^	0.48 ± 0.13 ^a^	0.29 ± 0.06 ^c^	0.26 ± 0.12 ^c^

**Table 4:** Flow cytometry analysis of cellular death using FITC-Annexin V/PI apoptosis detection at 48 h of incubation, where different letters (a, b, c) in the same column indicate significant differences between means (*p* ≤ 0.05) according to Tukey’s test; the values after ± represent the standard deviation of the mean.

Treatments	Healthy Cell (%)	Early Apoptosis (%)	Late Apoptosis (%)	Necrosis (%)
Control (nontreated)	89.9 ± 6.0 ^a^	9.1 ± 3.5 ^a^	0.7 ± 0.5 ^a^	0.3 ± 0.5 ^a^
Pure hexaconazole	47.0 ± 3.5 ^b^	49.1 ± 5.6 ^b^	3.2 ± 1.5 ^a^	0.7 ± 0.3 ^a^
Pure dazomet	4.2 ± 2.0 ^c^	10.2 ± 4.5 ^a^	85.5 ± 5.5 ^b^	0.1 ± 0.4 ^a^
CHEN	56.4 ± 5.5 ^b^	28.7 ± 2.0 ^c^	4.7 ± 2.3 ^a^	0.3 ± 0.5 ^a^
CDEN	34.5 ± 4.0 ^d^	52.4 ± 3.0 ^b^	12.2 ± 1.5 ^c^	0.9 ± 0.2 ^a^

**Table 5:** Skin reaction scores of (1) the initial test (one rabbit) and (2) the confirmatory test (two rabbits) upon removal of patch, where time indicates the time after the removal of a patch and 0 h indicates the immediate observation upon removal of a patch.

**(1) Initial Response**							
**Time (h)**	**CHEN**				**CDEN**		
	**Corrosion**	**Irritation**			**Corrosion**	**Irritation**	
		**Erythema**	**Edema**			**Erythema**	**Edema**
0	No corrosive lesion observed	1	1		No corrosive lesion observed	1	0
1	No corrosive lesion observed	1	0		No corrosive lesion observed	1	0
24	Brownish line lesion seen (5 mm length)	1	0		No corrosive lesion observed	2	0
48	-	-	-		Slight erosion on the skin	1	0
**(2) Confirmatory response**							
**Time (h)**	**Rabbit No.**	**CHEN**			**CDEN**		
		**Corrosion**	**Irritation**		**Corrosion**	**Irritation**	
			**Erythema**	**Edema**		**Erythema**	**Edema**
0	1	No corrosive lesion observed	1	1	No corrosive lesion observed	0	0
	2	No corrosive lesion observed	0	0	No corrosive lesion observed	1	1
1	1	No corrosive lesion observed	1	0	No corrosive lesion observed	0	0
	2	No corrosive lesion observed	1	1	No corrosive lesion observed	1	0

**Table 6:** Skin corrosion and irritation category and subcategories according to the Globally Harmonized System of Classification and Labelling Chemicals (GHS).

**Corrosion**	
Categories	Criteria
Category 1	Destruction of skin tissue in which necrosis of epidermis was observed in at least one tested animal after ≤4 h of exposure
Subcategory 1A	Skin corrosion in at least one tested animal at an exposure of ≤3 min upon removal of the test patch (observation period: ≤1 h)
Subcategory 1B	Skin corrosion in at least one tested animal at an exposure of >3 min and ≤1 h upon removal of the test patch (observation period: ≤14 days)
Subcategory 1C	Skin corrosion in at least one tested animal at an exposure of >1 h and ≤4 h upon removal of the test patch (observation period: ≤14 days)
**Irritatio**n	
Categories	Criteria
Irritation (Category 2)	Erythema and edema with a score of ≥2.3 and ≤4.0 in at least 2 out of 3 tested animals after 24, 48 and 72 h after removal of the test patch.
Mild irritation (Category 3)	Erythema and edema with a score of ≥1.5 and ≤2.3 in at least 2 out of 3 tested animals after 24, 48 and 72 h after removal of the test patch.
